# A Century of Change: Unraveling the Impact of Socioeconomic/Historical Milestones on Age at Menarche and Other Female Reproductive Factors in Japan

**DOI:** 10.2188/jea.JE20230155

**Published:** 2024-08-05

**Authors:** Madoka Iwase, Yukari Taniyama, Yuriko N. Koyanagi, Yumiko Kasugai, Isao Oze, Norikazu Masuda, Hidemi Ito, Keitaro Matsuo

**Affiliations:** 1 Division of Cancer Epidemiology and Prevention, Aichi Cancer Center, Nagoya, Japan; 2 Department of Breast and Endocrine Surgery, Nagoya University Hospital, Nagoya, Japan; 3 Division of Cancer Information and Control, Aichi Cancer Center, Nagoya, Japan; 4 Division of Descriptive Cancer Epidemiology, Nagoya University Graduate School of Medicine, Nagoya, Japan; 5 Department of Breast and Endocrine Surgery, Nagoya University Graduate School of Medicine, Nagoya, Japan; 6 Division of Cancer Epidemiology, Nagoya University Graduate School of Medicine, Nagoya, Japan

**Keywords:** reproductive factors, menarche, parity, breast feeding, secular change

## Abstract

**Background:**

Reproductive factors, such as age at menarche, are known to be associated with disease risk, but data on trends in these factors in Japan are limited. In this study, we investigated secular trends in reproductive factors and explored their potential association with socioeconomic and historical events.

**Methods:**

We conducted a retrospective analysis of 62,005 Japanese women born between 1890 and 1991 using a survey conducted over 25 years. Trends in reproductive factors were analyzed using linear and joinpoint regression models, and their associations with major historical events involving Japan were evaluated.

**Results:**

We found that the age at menarche showed a significant downward trend (*P* < 0.001) over the century. Three joinpoints were identified, in 1932 (15.23 years old), 1946 (13.48 years old), and 1959 (12.71 years old), which indicated that average age at menarche decreased by approximately 0.8% per year between 1932 and 1946, and then by 0.4% per year between 1946 and 1959, both of which were statistically significant. However, after 1959, age of menarche remained stable. Analyses of other reproductive factors found significant changes, including a decrease in parity and the number of babies breastfed, and an increase in age at first birth.

**Conclusion:**

Age at menarche showed a long-term downward trend in Japan, with significant change points in annual percent change. Other factors showed secular changes in trends as well. These change points were observed at the same time as historical events, namely wars and economic development, suggesting that socioeconomic and environmental changes at the population level affect reproductive factors in females.

## INTRODUCTION

Empirical evidence has established age at menarche as a significant factor in disease risk. Specifically, early menarche has been identified as a risk factor for breast cancer^1^ and all-cause mortality.^2^ In recent years, an emerging body of literature has documented a secular trend in decreasing age at menarche across various geographical regions and countries worldwide.^3^^–^^5^ Nonetheless, substantial variation exists in the reported rates of decrease, as well as in the timing of these shifts.^6^^–^^14^ To date, however, few of these reports have employed observation periods of up to 100 years, with the average being 50–60 years and some as short as 10 years.

These observed disparities may be attributed to an array of factors, including study design, genetic influences, and environmental determinants. These trends may exhibit distinct characteristics in Japan, an insular nation characterized by a predominantly mono-ethnic population and potentially divergent genetic and environmental factors to those in Western countries, but little long-term observational research has examined changes in menarcheal age within this context. Furthermore, there is a dearth of literature investigating the temporal dynamics of these changes and the etiological factors driving them.

The primary objective of the present study was to comprehensively investigate secular trends and changes in age at menarche and other female reproductive factors within a large-scale Japanese population over an extended temporal scale, encompassing approximately 100 years. Concurrently, the study endeavored to identify key time points temporally associated with these changes and to examine the possibility that these events were causally or otherwise associated with them. We speculated that a better understanding of these issues might contribute to our understanding of the development and mechanisms of hormone-related diseases, in particular, and in turn contribute to the development of health promotion strategies.

## METHODS

### Study population

We utilized data from the Hospital-based Epidemiologic Research Program at Aichi Cancer Center (HERPACC), which was conducted between 1988 and 2013. Details of the HERPACC study have been reported elsewhere.^15^^–^^17^ In brief, the study recruited first-visit outpatients at Aichi Cancer Center Hospital in Japan, and requested them to complete a lifestyle information questionnaire prior to their initial medical examination. The disease status of the participants was unknown at the time of enrollment, and their medical records were subsequently reviewed for up to 1 year following their first visit to determine their cancer status. Participants who were not diagnosed with cancer during the specified window period (3 months before the first visit to 1 year after the first visit) were classified as non-cancer.

The study was approved by the Institutional Ethics Committee at Aichi Cancer Center, and written informed consent was obtained from all participants.

### Variables and covariates

The primary outcome in this study was age at menarche. Additionally, we also evaluated several other reproductive factors as supplementary analyses, including parity, number of babies breastfed, age at first birth, and age at menopause. Information regarding these variables was obtained through self-administered questionnaires. All responses were provided in whole numbers as continuous variables. Participants with missing information for any variable were excluded from the respective analysis.

The questionnaire was also used to collect year of birth, which was validated using health insurance data. To account for the potential influence of economic status on the reproductive factors of interest, we utilized annual per capita data for gross domestic product (GDP) in Japan from the Maddison Project Database 2018.^18^

### Statistical analysis

To assess secular changes in reproductive factors by birth year, we calculated the average values and 95% confidence intervals (CIs) for each outcome variable, stratified by the participants’ birth year. We employed joinpoint regression analysis (Joinpoint Trend Analysis Software version 4.7.0.0; United States National Cancer Institute, Bethesda, MD, USA)^19^ to estimate trends in the average values of each variable by birth year, with a maximum of 3 joinpoints (4 line segments). In essence, joinpoint regression is a statistical method used to characterize changes in trends across successive time segments. It identifies the best-fitting linear model for each segment and describes the trend as “increasing” or “decreasing” when the slope of the trend is statistically significantly different from zero, or otherwise uses the terms “stable” or “level”. The results of trends are presented as annual percent change (APC) and average annual percent change (AAPC), with two-sided statistical significance (*P* < 0.05).

For evaluation of age at menarche, all subjects were included. Conversely, in the supplementary analyses, analysis of the number of deliveries, number of babies breastfed, and age at first birth was restricted to those who were at least 50 years old at the time of participation, while age at menopause was restricted to those who were at least 60 years old and known to have experienced natural menopause at the time of participation.

All other statistical analyses were performed using STATA software version 15.1 (Stata Corp., College Station, TX, USA).

## RESULTS

A total of 62,005 women without any cancer at baseline were included. Average age at first visit for all participants was 46.7 (standard deviation, 13.1) years, and year of birth ranged from 1890 to 1991. Participants characteristics and distribution of 10-year age categories at baseline are presented in Table 1 and eFigure 1.

**Table 1.** Characteristics of participants

		*N*	%
Total		62,005	

Age at first visit, years, mean (SD)		46.7 (13.1)	
Age at first visit, 10-year category			
	<20	424	0.7%
	20–29	6,104	9.8%
	30–39	11,655	18.8%
	40–49	18,820	30.4%
	50–59	14,104	22.7%
	60–69	8,021	12.9%
	70–79	2,789	4.5%
	80–89	84	0.1%
	≥90	4	<0.1%
Year of birth	1890–1899	3	<0.1%
	1900–1909	67	0.1%
	1910–1919	825	1.3%
	1920–1929	4,328	7.0%
	1930–1939	9,847	15.9%
	1940–1949	18,713	30.2%
	1950–1959	13,697	22.1%
	1960–1969	9,400	15.2%
	1970–1979	4,487	7.2%
	1980–1989	632	1.0%
	1990–1999	6	<0.1%
BMI,^*^ median (range)		21.1 (12.8–46.0)	
Smoking^*^	Never	7,935	77.9%
	Ever	2,257	22.1%
Drinking^*^	Never	5,865	57.4%
	Ever	4,338	42.5%
	Unknown	16	0.2%

BMI, body mass index; SD, standard deviation.

^*^Data obtained from the questionnaire at the first visit.

### Age at menarche

**Figure 1.**
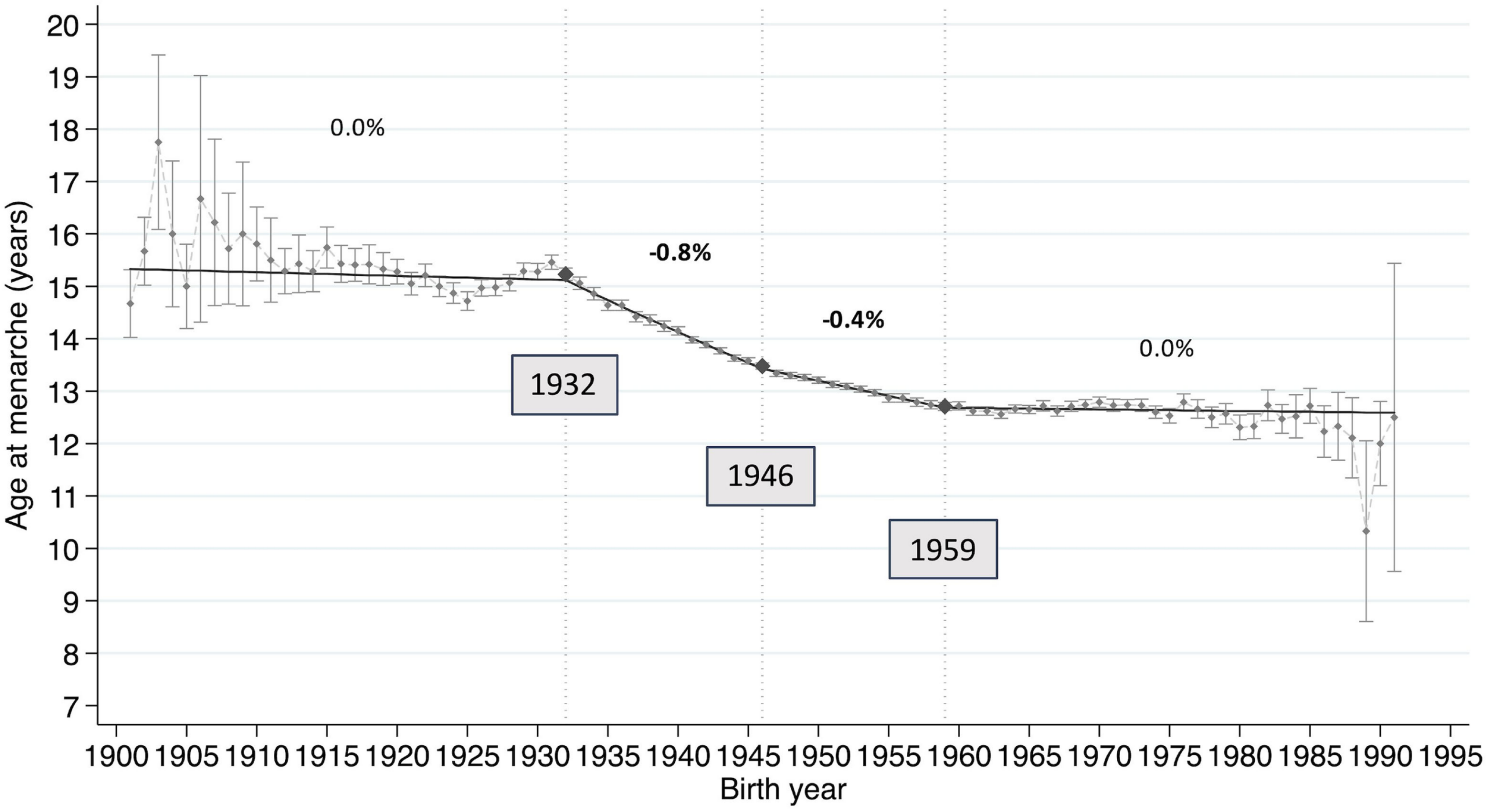
Secular trends in age at menarche and joinpoints. The trend in average age at menarche among Japanese women according to birth year showed a significant long-term downward trend. Joinpoint regression analysis revealed three joinpoints, in 1932, 1946, and 1959.

Of the total 62,005 participants, 474 had no information about age at menarche and were excluded from analysis, resulting in a final sample of 61,531 participants for analysis of age at menarche (eFigure 2). Figure 1 shows the trends from 1901 to 1991 in average age at menarche for each birth year, using a linear model of joinpoint regression analysis and the significant joinpoint years of APC. Trends in outcomes by birth year as determined by these joinpoint regression analyses are presented in Table 2. A downward trend in age at menarche was observed, with an AAPC of −0.219% (95% CI, −0.255 to −0.183%). and three joinpoints of APC. These joinpoints were identified in 1932 (15.23 years old), 1946 (13.48 years old), and 1959 (12.71 years old). Age at menarche began to decrease by approximately 0.8% per year between 1932 and 1946, and then by 0.4% per year between 1946 and 1959, both of which were statistically significantly different from zero. Conversely, age of menarche remained stable after 1959.

**Table 2.** Trends of average of age at menarche by birth year with joinpoint analyses

	Period	APC^a^	(95% CI)	*P*-value^b^
Trend 1	1901–1932	−0.045	(−0.13 to 0.039)	0.29
Trend 2	1932–1946	**−0.843**	(−0.914 to −0.773)	<1.00E-10
Trend 3	1946–1959	**−0.436**	(−0.502 to −0.37)	<1.00E-10
Trend 4	1959–1991	−0.024	(−0.072 to 0.024)	0.317


		AAPC^a^	(95% CI)	*P*-value^b^

Total		**−0.219**	(−0.255 to −0.183)	<1.00E-10

AAPC, average annual percent change; APC, annual percent change; CI, confidence interval.

^a^Values in bold character show statistically significantly different from zero.

^b^*P*-values <0.05 are regarded as significant (two-sided, calculated using a *t*-test).

### Other reproductive factors

The consort diagram depicting eligible participants for analysis of each variable is shown in eFigure 2. In total, 24,682 participants were analyzed for parity, 19,923 for number of babies breastfed, 22,283 for age at first birth, and 9,080 for age at menopause. The years of observation for three these reproductive factors (parity, number of babies breastfed, and age at first birth) ranged from 1901 to 1962, while the years of observation for age at menopause ranged from 1901 to 1952. In the analysis of parity, the AAPC was −1.6% (95% CI, −1.802 to −1.353%). Joinpoint analysis revealed 2 joinpoints (1928, 1938), with a 3.8% decrease per year from 1901 to 1928, followed by a 0.7% increase between 1928 and 1938. In the analysis of the number of babies breastfed, the AAPC was −1.6% (95% CI, −2.020 to −1.189%), and 3 joinpoints (1928, 1948, 1952) were identified, with a 4.1% decrease in APC between 1901 and 1928 and a 5.7% increase between 1948 and 1952. In the analysis of age at first birth, the AAPC was 0.4% (95% CI, 0.094 to 0.757%) for the entire period, with a statistically significant increase of 0.3% in APC between 1906 and 1932, a decrease of 0.1% between 1932 and 1944, and an increase of 0.2% between 1944 and 1962. In the analysis by age at menopause, the AAPC was 0.1% (95% CI, 0.016 to 0.146%), and 2 joinpoints (1923, 1926) were identified, with a 0.1% increase in APC between 1926 and 1952 (eTable 1 and eFigure 3).

### Comparison with social and economic conditions

**Figure 2.**
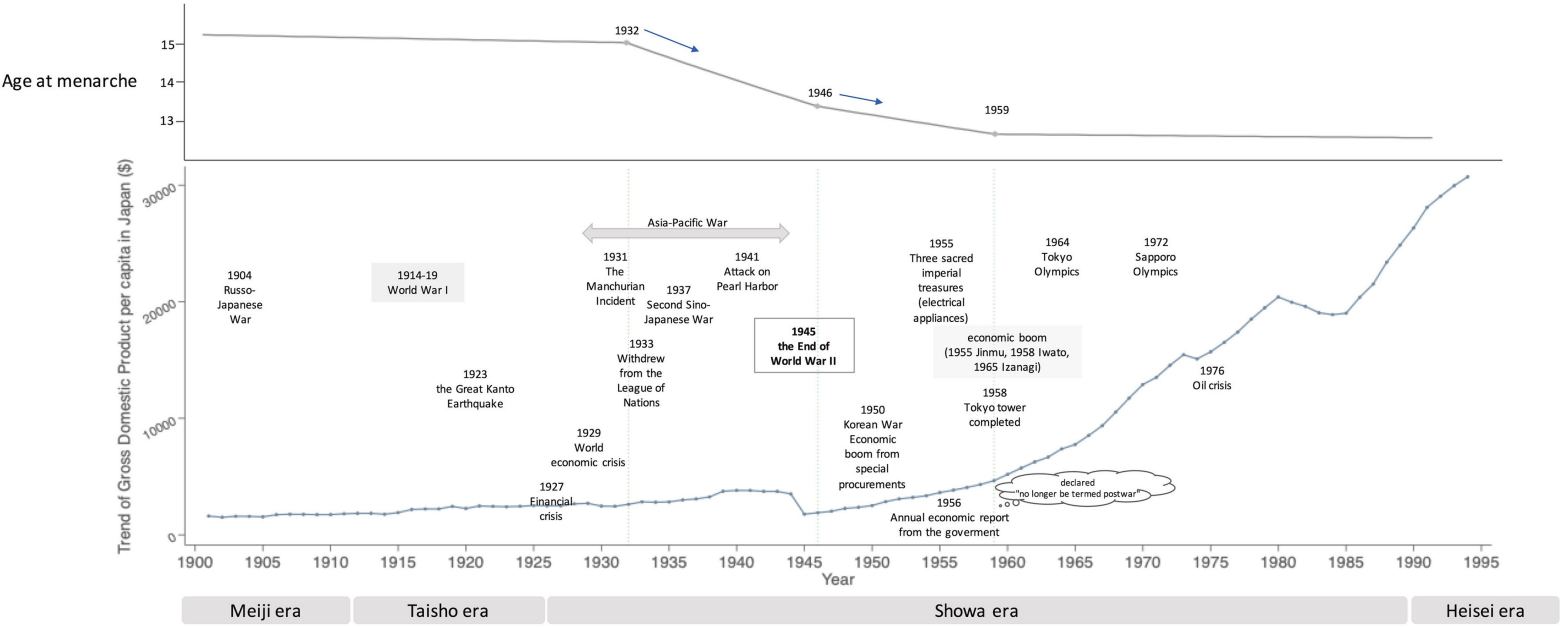
Trends in age at menarche and GDP per capita with historical events over a century in Japan. Secular trends in age at menarche are shown with the change in GDP per capita and historical events over a century in Japan. The three joinpoints in age at menarche, in 1932, 1946, and 1959, coincided with historical events as well as economic growth.

Figure 2 shows the evolution of Japan’s historical social conditions and GDP. The trend in GDP in Japan showed a gradual increase until late in World War II and then a temporary and marked dip after the end of the war. GDP had almost fully recovered by around 1960, and rapid and dramatic economic growth was achieved thereafter. Examining the trend in age at menarche and these historical events reveals that the first joinpoint in age at menarche was 1932, coinciding with the time when Japan invaded Manchuria and initiated the conflict that later escalated to World War II. The next joinpoint of age at menarche was 1946, which coincided with the end of World War II. Furthermore, the latest joinpoint of age at menarche occurred roughly at the time when Japan regained its diminished national strength, or what might be termed the normalization of its economic and social position, after its defeat in the war, and entered into an era of rapid economic growth.

Parity and number of babies breastfed showed clear numerical changes before the period of the Japanese invasion of China (eFigure 3A, eFigure 3B). Age at first birth underwent a significant shift after the Russo-Japanese War, and while it briefly decreased following World War II due to the baby boomer generation, it has consistently trended towards older ages ever since (eFigure 3C). In contrast, age at menopause showed no clear association with these historical events (eFigure 3D).

## DISCUSSION

In this study, we demonstrated that age at menarche in Japan significantly decreased by 0.2% per year over approximately 100 years, or in other words by about 0.30 years per decade. Moreover, the change in this curve has not been constant, but has rather demonstrated significant change points and trends. Each significant change point appeared to coincide with major social and historical events in Japan, which may in turn imply that these major events and changes in society had a plausible impact on individual reproductive factors.

Consistent with our findings, numerous previous studies in various countries and ethnicities have reported a downward trend in age at menarche. For example, a large multicenter study involving participants from nine European countries born between 1915 and 1964 reported that mean age at menarche decreased by 44 days per 5-year birth cohort. Although some differences between countries were observed, age at menarche decreased over time in all nine countries.^5^ Similarly, studies in Asian countries, such as China^3^ and Korea,^6^ have also reported declining trends in age at menarche, at 0.51 years and 0.726 years per decade, respectively. Our results support these observed declining tendencies.

Various factors have been proposed to explain the downward trends or changing trend curves in age at menarche. Improved nutrition and socioeconomic circumstances are widely believed to contribute to the decline to some extent.^4^ Several studies have reported that body weight, including body mass index, influences the onset of menarche^20^^–^^22^ and is negatively associated with age at menarche.^3^^,^^23^^–^^25^ Furthermore, factors such as breastfeeding and consumption of animal fat or protein, carbohydrates, soybean products, and fortified products (with supplements like dietary fiber, calcium, and vitamin D) have also been suggested to potentially influence the timing of female sexual maturation.^3^^,^^22^

Socioeconomic circumstances also play a role, as girls with higher socioeconomic status have been reported to experience earlier menarche.^26^ Some studies have found that girls from urban areas have a relatively higher age at menarche than those from rural areas, indicating that area of residence or geographic region, which possibly reflects socioeconomic environment, is also associated with age at menarche.^3^^,^^7^^,^^8^^,^^10^ In the present study, we propose that decreasing age of menarche can also be understood from another viewpoint; namely, the relationship between trend points in age at menarche and social conditions of the time period (Figure 2). Under this viewpoint, the joinpoints of age at menarche appeared to coincide with the joinpoints of Japanese history.

Such changes in reproductive factors and lifestyles are often thought to be due solely to individual biological factors, but historical background and social conditions may affect socioeconomic status at the population level, which may in turn result in changes in reproductive factors at the individual level. Examining such population-based lifestyle changes in relation to age at menarche is a new perspective. Our findings suggest that social emergencies, such as war, can profoundly affect population reproductive factors that are not simply driven by the economic or nutritional status of individuals, potentially via effects on medical administration and public health. Gomula and Koziel reported an increase in age at menarche during years of economic crisis. Social emergencies may encompass not only wars but also pandemics caused by infectious diseases, like the current coronavirus disease 2019 (COVID-19) crisis. A similar phenomenon was reported by DeWitte et al, who indicated that the age of menarche changed before and after the Black Death.^27^ As these reproductive factors as a surrogate indicator of lifetime estrogen exposure are known to be associated with numerous diseases, it is crucial to recognize that social events can influence human biological events and indirectly affect the burden of disease. Amid the ongoing COVID-19 pandemic, we are experiencing significant impacts on our society’s healthcare system, insurance administration, and economic activities.^28^^–^^30^ It is possible that economic factors of individual households affected age at first menarche, but it is also possible that the effects of social events, such as pandemics and wars, on reproductive factors are derived via complex and composite factors, such as the economy, nutritional status, and welfare and medical environment conditions, triggered by those events. This study examined the significance of social changes that may be the major trigger for such changes, not just a single factor. It is essential to consider the possibility that such social events may also affect biological changes in humans, warranting further research in this area.

In our study, we also observed significant changes in other reproductive factors: parity, number of babies breastfed, and age at first birth. Intriguingly, these factors exhibited distinct shifts around the periods preceding major historical events, specifically before the Japanese invasion of China and after the Russo-Japanese War. For instance, both parity and the number of breastfeeding instances demonstrated clear changes before the Japanese invasion of China. Additionally, age at first birth saw a notable transition after the Russo-Japanese War, and despite a temporary decrease post-World War II, likely influenced by the baby boomer generation, it has consistently moved towards older ages in the subsequent years.

These observations underscore the profound influence of historical and societal upheavals on reproductive behaviors. Specifically, such events, coupled with the socioeconomic changes that accompany them, can lead to shifts in societal norms, healthcare practices, and individual choices, all of which can impact reproductive trends. However, it is worth noting that age at menopause did not exhibit a similar pattern of change in association with these historical events as the other factors. This suggests that while early and mid-reproductive life events might be more susceptible to societal and historical influences, such factors have little effect on the later stages of reproductive life, such as age at menopause. This lack of influence might be because reproductive factors in the later stage of life are influenced by a complex interplay of various individual and environmental factors, rather than being solely governed by major societal shifts.

Several limitations of the present study should be acknowledged. First, the information was collected using self-administered questionnaires, which might have led to inaccuracies. However, participants in our study answered the questionnaires systematically without knowing the study topic, which may have minimized recall bias. We confirmed the influence of recall bias by analyzing the mean age at first menarche within the same birth year cohort across different survey years. For instance, for participants born between 1920–24, mean age at menarche reported in the 1988 survey was 15.01 years, while in the 2000 survey, it was 15.2 years. Similarly, for those born between 1940–44, the values were 13.8 years (1988 survey) and 13.9 years (2000 survey). For the cohort born between 1965–69, the reported mean ages were 12.6 years (1988 survey) and 12.7 years (2000 survey). Based on these findings, recall bias had minimal influence on our results. Second, our data were obtained from a single institution. Despite this limitation, we propose that it is valuable to observe women’s health over such a long term under the same regional circumstances, with quality-control measures provided by well-trained investigators. Third, the current state of age at menarche, whether it has reached its limit or is still decreasing, remains controversial, as some European studies indicate that the steady decline in age at menarche appears to have stopped^5^^,^^9^^,^^11^^,^^12^ while others report a further decrease after stabilization.^3^^,^^4^^,^^6^^,^^8^^,^^13^ Since all women in our study were born before 2000, our study could not reveal the most recent trends in age at menarche or other female reproductive factors. Further research is needed to clarify these recent trends. Additionally, obtaining accurate information on female reproductive factors other than age at menarche requires assessment after reproductive age, so the trend changes may seem too short to judge clearly due to the limited number of eligible women and an insufficient duration of observation of the period of fertility.

Despite these limitations, our study has several strengths. To our knowledge, this large-scale study of secular trends in age at menarche and other female reproductive factors has the longest observation period among similar studies in Japan. Our finding of region-specific trends is particularly meaningful, given that these factors may be influenced not only by genetics but also by social background or environmental situation, which can vary among countries and ethnicities. Furthermore, investigating trend changes might help identify the causes of these changes, which might in turn contribute to disease prevention and health promotion efforts.

In conclusion, our study revealed a long-term downward trend in age at menarche in Japan. It also indicated that the APC trend change points were associated with historical events, such as wars and economic development, suggesting that the population-based socioeconomic environment is related to female reproductive factors. Further studies are warranted to clarify the relationship between environmental and reproductive factors and to reveal more recent trends to better understand and address their potential impact on public health.

## SUPPLEMENTARY MATERIAL

eFigure 1. Distribution of age categories at participation according to birth year.

eFigure 2. Subject selection for each analysis among the whole subject.

eFigure 3. Secular trends of other female reproductive factors and joinpoints. The trend in average (**A**) parity, (**B**) number of breastfeeding, (**C**) age at first delivery, and (**D**) age at menopause among Japanese women according to birth year. For each analysis, years in square indicate joinpoints revealed in the joinpoint regression analysis. Annual percent change (APC) during each joinpoint is shown. Bold number indicates APC with *P*-value <0.05. Parity showed a significant downward trend until 1928, followed by a small increasing trend until 1938 and a stable trend afterward. Breastfeeding showed a significant downward trend until 1928. After 20 years of stable trend, four years of an increasing trend, in parallel with the baby boom after World War II. Age at the first delivery showed a significant increase from 1906 to 1932, followed by 8 years of significant decrease and increase afterward. Age at menopause showed a significant small increase trend after 1926. Numbers in bold are statistically significant with a corresponding number of APC.
