# Supplementary material for: A Century of Change: Unraveling the Impact of Socioeconomic/Historical Milestones on Age at Menarche and Other Female Reproductive Factors in Japan
Source: J Epidemiol. 2024 Aug 5;34(8):387–92. doi: 10.2188/jea.JE20230155 (PMC11230879; doi:10.2188/jea.JE20230155)
Supplement: Supplementary file 1 [file je-34-387-s001.pdf]

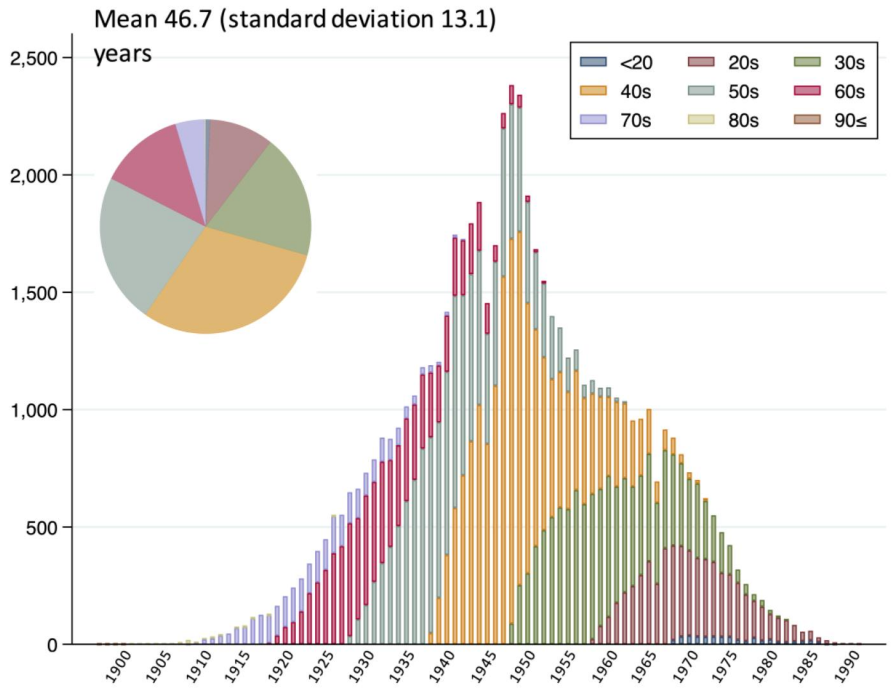

eFigure 1. Distribution of age categories at participation according to birth year.

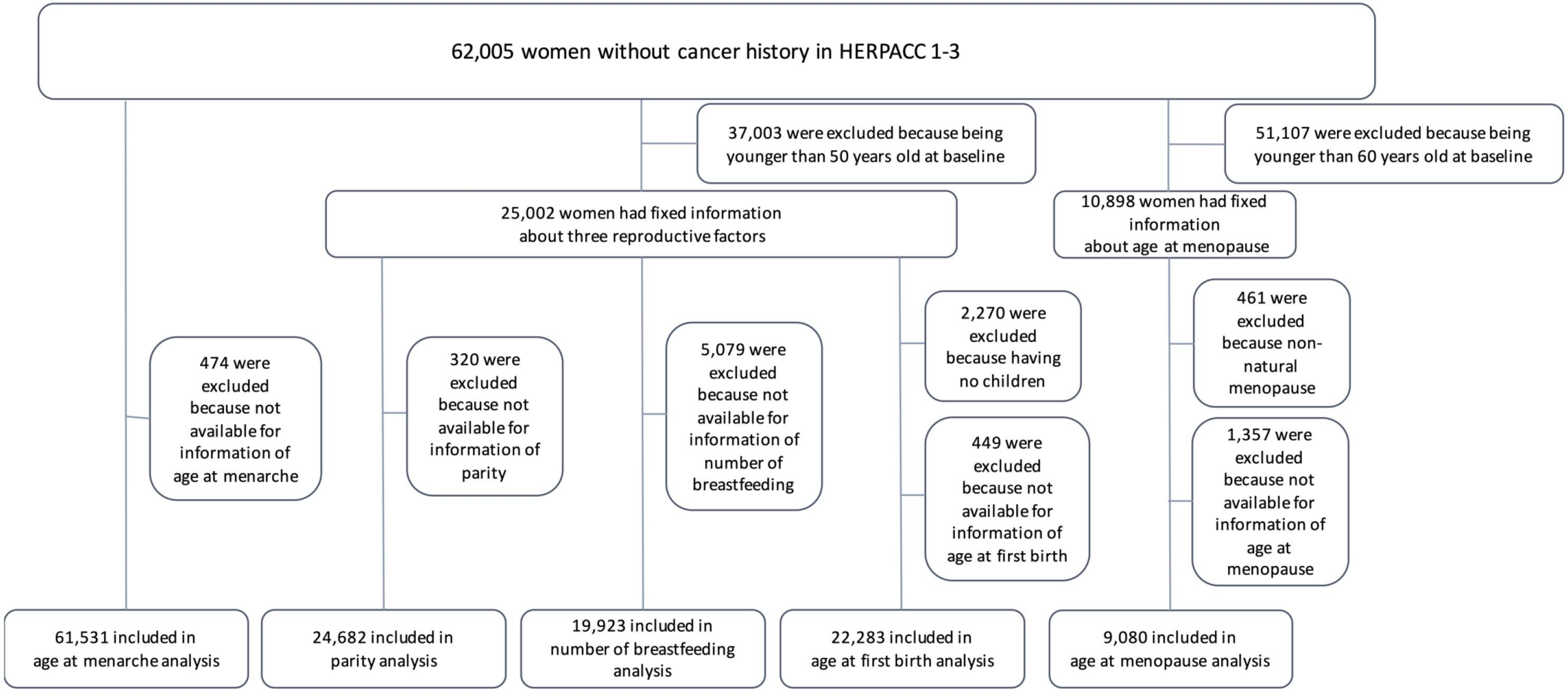

eFigure 2. Subject selection for each analysis among the whole subject.

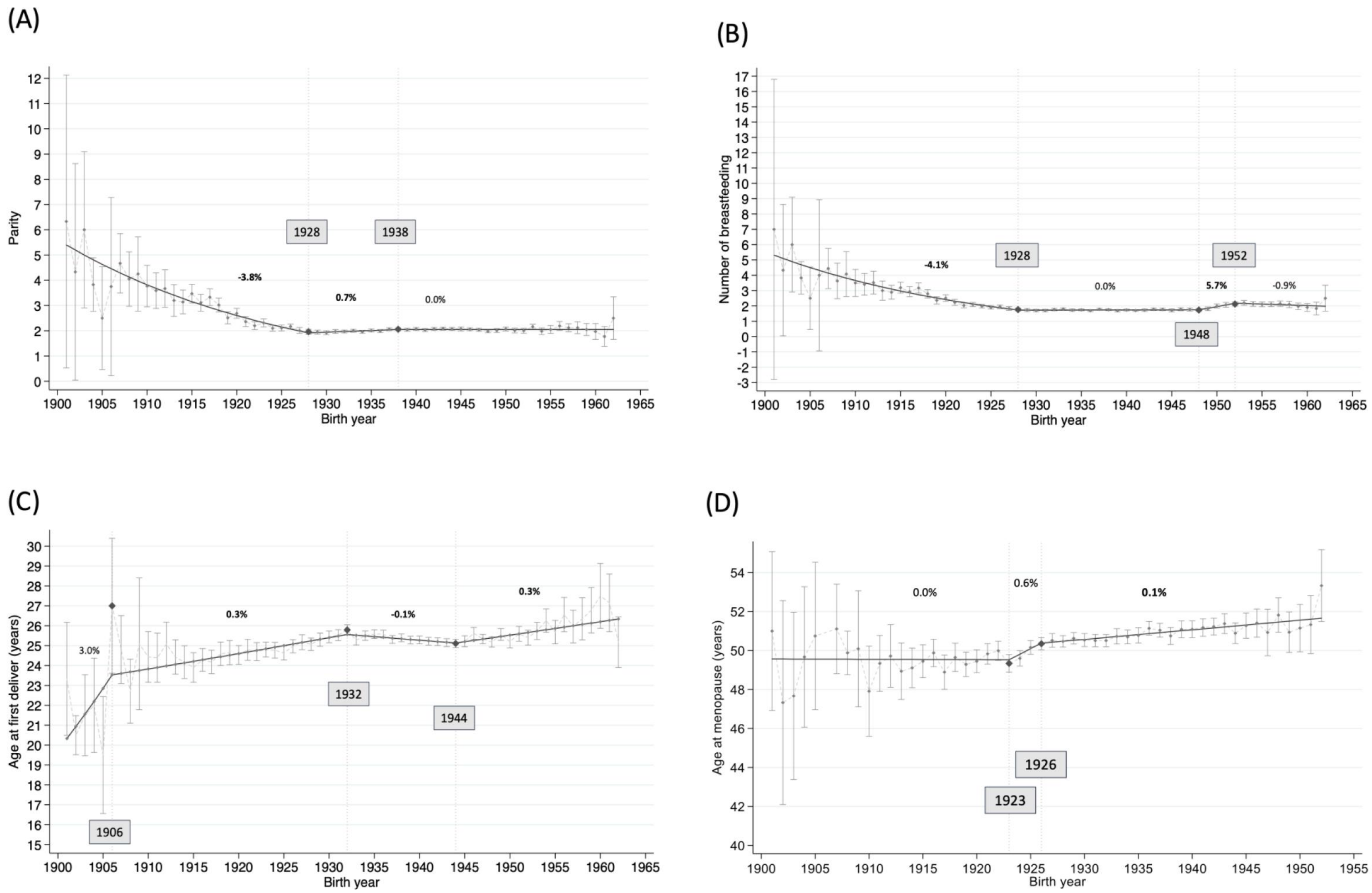

eFigure 3. Secular trends of other female reproductive factors and joinpoints. The trend in average (**A**) parity, (**B**) number of breastfeeding, (**C**) age at first delivery, and (**D**) age at menopause among Japanese women according to birth year. For each analysis, years in square indicate joinpoints revealed in the joinpoint regression analysis. Annual percent change (APC) during each joinpoint is shown. Bold number indicates APC with *P*-value <0.05. Parity showed a significant downward trend until 1928, followed by a small increasing trend until 1938 and a stable trend afterward. Breastfeeding showed a significant downward trend until 1928. After 20 years of stable trend, four years of an increasing trend, in parallel with the baby boom after World War II. Age at the first delivery showed a significant increase from 1906 to 1932, followed by 8 years of significant decrease and increase afterward. Age at menopause showed a significant small increase trend after 1926. Numbers in bold are statistically significant with a corresponding number of APC.
